# The Role of Inflammatory and Nutritional Indices in Postmenopausal Osteoporosis: A Retrospective Study

**DOI:** 10.3390/jcm13247741

**Published:** 2024-12-18

**Authors:** Busra Demir Cendek, Burak Bayraktar, Mehmet Alican Sapmaz, Ayse Ecenaz Yıldırım, Mujde Can Ibanoglu, Yaprak Engin Ustun

**Affiliations:** 1 Department of Obstetrics and Gynecology, Health Sciences University Etlik Zubeyde Hanim Maternity, Teaching and Research Hospital, 06010 Ankara, Turkey; drmujdecan@gmail.com (M.C.I.); ustunyaprak@yahoo.com (Y.E.U.); 2 Department of Obstetrics and Gynecology, Republic of Turkey Ministry of Health Ankara Etlik City Hospital, 06710 Ankara, Turkey; dr.alicansapmaz@hotmail.com (M.A.S.); aecenazzz@gmail.com (A.E.Y.); 3 Department of Obstetrics and Gynecology, Division of Perinatology, Health Sciences University Etlik Zubeyde Hanim Maternity, Teaching and Research Hospital, 06010 Ankara, Turkey; drburakbayraktar@gmail.com; 4 Department of Obstetrics and Gynecology, Division of Perinatology, Republic of Turkey Ministry of Health Ankara Etlik City Hospital, 06710 Ankara, Turkey

**Keywords:** osteoporosis, osteopenia, postmenopausal women, nutrition, inflammation, bone mineral density

## Abstract

**Background**: Postmenopausal osteoporosis is characterized by impaired bone metabolism, inflammation, and nutritional deficiencies. This study aimed to evaluate the potential of inflammatory and nutritional markers in identifying decreased bone mineral density (BMD) in postmenopausal women. **Methods**: This cross-sectional study retrospectively analyzed postmenopausal women from January 2018 and December 2023. A total of 368 women were divided into three groups based on T-scores: 61 women with osteoporosis (T-score ≤ −2.5), 153 women with osteopenia (−1 > T-score > −2.5), and 154 women with normal BMD (T-score > −1). Inflammatory and nutritional biomarkers included the neutrophil/lymphocyte ratio (NLR), platelet/lymphocyte ratio (PLR), monocyte/lymphocyte ratio (MLR), systemic immune-inflammation index (SII), systemic inflammation response index (SIRI), pan-immune inflammation value (PIV), geriatric nutritional risk index (GNRI), triglycerides, total cholesterol, and body weight index (TCBI), prognosis nutritional index (PNI), hemoglobin, albumin, lymphocyte, and platelet (HALP) score, 25-OH Vitamin D level, Na, K, Ca, Mg, and their ratios. **Results**: The GNRI was significantly lower in the osteoporosis group compared to the control group. The NLR, PLR, SII, SIRI, PIV, TCBI, PNI, and HALP were similar between the groups. The GNRI and TCBI showed a positive correlation with T-scores. The Mg level was lower in the osteoporosis group than in the control group and osteopenia group, and the Na/Mg ratio was higher. Additionally, the Ca/Mg ratio was lower in the osteoporosis group than in the control group. The T-score was positively correlated with Mg and Ca/Mg, while the Na/Mg ratio showed a significant negative correlation. Vitamin D, other minerals, and their ratios did not show significant differences between the groups. **Conclusions**: Our findings suggest that the GNRI could serve as a useful indicator for assessing bone health and the risk of osteoporosis. Furthermore, maintaining appropriate levels of Mg and balanced Na/Mg and Ca/Mg ratios appears crucial for BMD.

## 1. Introduction

The climacteric period marks a significant phase in a woman’s life, characterized by substantial physiological and hormonal transformations [1]. Recent advancements in living conditions have improved diagnostic and treatment procedures, resulting in a significant extension of the human lifespan. Furthermore, there has been a rise in the global population of women experiencing menopause. Based on the data in Turkey, it is estimated that women will live for around 81 years [2]. During this time, they will spend around 27 years, which is one-third of their lives, in the postmenopausal stage. During this period, they may experience health problems specific to this stage of life.

Menopause is characterized by a decrease in estrogen secretion, resulting in reduced bone density and the potential acceleration of severe osteoporosis development [3]. Postmenopausal osteoporosis (PMOP) is a chronic and systemic disorder of bone metabolism, characterized by the loss of bone mass, microstructural degradation, and increased susceptibility to fragility fractures [4,5]. The causes of osteoporosis and osteopenia are complex and involve multiple factors, including genetic variations, endocrine influences, lifestyle choices such as physical activity and dietary patterns, and inflammation, particularly the deficiency of minerals resulting from decreased dietary intake and impaired nutrient absorption, which is believed to significantly contribute to the development of osteopenia or osteoporosis, as well as to its prevention [6,7]. Assessing inflammation and nutritional status is a considerable challenge. After menopause, after estrogen secretion stops, women experience a persistent state of low-grade inflammation in their bodies [8,9]. The field of bone immunology suggests that inflammatory mediators have a significant impact on the development of osteoporosis [9]. Osteoporosis or fractures have been linked to many inflammatory biomarkers that can be obtained from complete blood counts (CBCs) [10,11,12,13,14,15]. The neutrophil/lymphocyte ratio (NLR), platelet/lymphocyte ratio (PLR), monocyte/lymphocyte ratio (MLR), systemic immune-inflammation index (SII), systemic inflammation response index (SIRI), and pan-immune inflammation value (PIV) are blood-cell indices obtained from a CBC that may provide a more accurate representation of the inflammatory condition of a disease compared to counting individual cells [10,11,12,13,14,15]. These have been studied before, but their reliability is questionable because they are nonspecific and give different results in different populations. The geriatric nutritional risk index (GNRI), triglycerides, total cholesterol, and body weight index (TCBI), prognosis nutritional index (PNI), and hemoglobin, albumin, lymphocyte, and platelet (HALP) score are new indices that reflect blood-based inflammation and nutrition [16,17]. These indices deserve further investigation in the context of osteoporosis due to their multi-parameter nature and their dual focus on inflammation and nutrition.

The majority of studies on the relationship between nutrition and bone health have focused on the functions of calcium, Vitamin D, and their combined effect. However, it has been demonstrated that other nutrients also have a significant impact. An example of a mineral that is in need of additional examination relative to its role in bone health is magnesium [18]. Magnesium is the fourth most abundant mineral in the body, 60% of which is stored in bone [19]. It has been observed that magnesium has a significant impact on bone metabolism through its influence on the activity of factors associated with bone formation and resorption [19,20]. Additionally, it exerts an impact on calcium metabolism and plays a role in the regulation of several hormones, such as the parathyroid hormone and calcitriol, which is the biologically active form of Vitamin D [20,21,22]. Consequently, magnesium may play a critical role in the diagnosis, prevention, and management of osteoporosis.

The incidence of PMOP is increasing due to the rapid aging of the population. This phenomenon has emerged as a significant societal health concern and imposes substantial economic costs [23,24]. The conventional method of diagnosing PMOP is primarily based on the utilization of dual-energy X-ray absorptiometry (DEXA) and the evaluation of bone mineral density (BMD) with the T-score [5]. Hence, there is a pressing need to find readily available and efficient biomarkers for the early detection, screening, and severity prediction of PMOP [25]. The objective of this study was to examine the correlation between systemic inflammation and nutritional indices (NLR, PLR, SII, SIRI, PIV, GNRI, TCBI, PNI, HALP), the 25-OH Vitamin D level, mineral levels (Na, K, Ca, Mg), mineral ratios (Na/K, Na/Ca, Na/Mg, K/Mg, Ca/K, Ca/Mg), and PMOP. Additionally, we sought to assess the potential of these indicators as markers for the early detection of decreased BMD in postmenopausal women.

## 2. Materials and Methods

This cross-sectional study was conducted at the Department of Obstetrics and Gynecology, Health Sciences University Etlik Zubeyde Hanim Maternity, Teaching and Research Hospital in Ankara, Turkey. It involved a retrospective analysis of medical records for 368 eligible postmenopausal women between January 2018 and December 2023. This study was conducted after ethical approval was obtained from the Health Sciences University Etlik Zubeyde Hanim Maternity, Teaching and Research Hospital Ethics Committee (approval no: 22.01.2024-01). Informed consent was not necessary due to the retrospective nature of this study, and it was waived by Health Sciences University Etlik Zubeyde Hanim Maternity, Teaching and Research Hospital Ethics Committee. The principles of the Declaration of Helsinki were applied in this study. Patient data were extracted from medical records and the hospital information management system.

A total of 368 women were categorized into three groups based on the T-scores. Group 1 comprised 61 women diagnosed with osteoporosis (referred to as the osteoporosis group, with a T-score ≤ −2.5). Group 2 included 153 women diagnosed with osteopenia (referred to as the osteopenia group, with a −1 > T-score > −2.5). Lastly, Group 3 comprised 154 women with normal BMD (referred to as the control group, with a T-score > −1). This study included patients diagnosed with menopause who underwent DEXA and blood tests to assess BMD and whose test results were available in the hospital’s electronic database. Women were excluded if they had received hormone therapy during the reproductive period, were using corticosteroids, had a history of malignancy or fracture, experienced immobility (e.g., prolonged bed rest or wheelchair dependence), or had systemic diseases such as diabetes, hypertension, kidney disease, liver disease, or depression. Additionally, individuals with incomplete medical records, DEXA, and laboratory results were also excluded.

The STRAW (Staging Reproductive Aging in Women) staging system was developed in 2001 to define reproductive aging based on menstruation patterns and follicle-stimulating hormone (FSH) levels. The staging system was revised in 2011 to incorporate additional parameters, including FSH, antral follicle count (AFC), the anti-mullerian hormone (AMH), and inhibin-B. This system divides a woman’s life into three main periods: the reproductive period, the menopausal transition period, and the postmenopausal period; the menopausal transition period and the postmenopausal period were further subdivided into seven phases—five phases before the final menstruation and two phases after [26]. The postmenopausal period, characterized by the STRAW +1 and +2 phases, begins after the final menstrual cycle and is subdivided into four clusters based on fluctuations in FSH and estradiol levels. Phase +1a encompasses the first year of amenorrhea following the final menstrual cycle. Phase +1b starts in the second year postmenopause and continues until FSH and estradiol levels stabilize. These two phases together typically last about two years. Phase +1c, defined by stable elevated FSH and reduced estradiol levels, spans approximately 3–6 years. Consequently, the early postmenopausal phase generally lasts 5–8 years [26].

Retrospective data collected included age, age of menopause, duration of menopause, gravida, parity, body mass index (BMI), STRAW score, blood tests, biochemical markers, and BMD values. Laboratory examinations and BMD assessments were conducted concurrently at the time of the patient’s admission. BMD was measured at the lumbar spine and femoral neck using dual-energy X-ray absorptiometry (DEXA) [STRATOS DR 2D-Fan Beam Whole Body Bone Densitometer, Monteux, France]. T-scores were calculated based on these measurements, and the lowest T-score from the lumbar spine or femoral neck was used for diagnostic classification, following WHO criteria [27].

Inflammatory and nutritional biomarkers calculated included the neutrophil/lymphocyte ratio (NLR), platelet/lymphocyte ratio (PLR), monocyte/lymphocyte ratio (MLR), SII = neutrophil × platelet/lymphocyte, SIRI = neutrophil × monocyte/lymphocyte, PIV = neutrophil count × platelet count × monocyte count/lymphocyte count, GNRI = 14.89 × serum albumin (g/dL) + 41.7 × (measured BW (kg)/ideal BW (kg)), TCBI = triglycerides (mg/dL) × total cholesterol (mg/dL) × body weight (BW) (kg)/1000, PNI = 10 × albumin (g/dL) + 0.005 × total lymphocyte count (per mm³), and HALP score = hemoglobin (g/L) × albumin (g/L) × lymphocyte count (/L)/platelet count (/L).

### Statistical Analysis

All statistical analyses were performed using IBM^®^ SPSS^®^ Statistics version 26.0. Variables were assessed for normality using visual methods (histograms, probability plots) and analytical techniques (Kolmogorov–Smirnov/Shapiro–Wilk tests). Levene’s test assessed the homogeneity of variances. Numerical data that did not show normal distribution were presented using medians and quartiles (Q1–Q3), the Kruskal–Wallis test was applied, and a post-hoc Mann–Whitney U test was used to determine which groups were significant. Numerical data that showed normal distribution were presented as means ± SD, the ANOVA test was applied, and post-hoc and pairwise differences were assessed using the Student’s *t*-test. Relationships between categorical variables were analyzed with the Chi-square test. Correlation coefficients and their significance were calculated using the Spearman test. The predictive capacity of various parameters was analyzed using receiver operating characteristic (ROC) curve analysis. A *p*-value of less than 0.05 was considered statistically significant.

## 3. Results

Demographic, clinical, and laboratory characteristics and the outcomes between the groups are shown in Table 1. Accordingly, maternal age was higher in the osteoporosis group than in the control group and osteopenia group (*p* < 0.001). There were no significant differences among the groups in terms of gravida, parity, and age of menopause. The BMI was higher in the control group compared to the osteoporosis group, and the duration of menopause was shorter in the control group than in the osteoporosis group (*p* = 0.029 and *p* = 0.001, respectively). According to STRAW staging, a higher proportion of individuals in the osteoporosis group (67.2%) were classified as Stage 2, compared to the osteopenia (53.6%) and control groups (44.2%) (*p* = 0.008). As expected, BMD progressively increased from the osteoporosis to the osteopenia and control groups (−2.98 ± 0.44 vs. −1.72 ± 0.46 vs. 0.21 ± 1.02, *p* < 0.001). The levels of albumin, total cholesterol, triglycerides, hemoglobin, neutrophils, lymphocytes, monocytes, and platelets were similar across the groups.

An examination of nutritional and inflammatory indexes, the 25-OH Vitamin D level, and minerals between the groups is shown in Table 2. The GNRI score was significantly lower in the osteoporosis group compared to the control group (116 ± 9.4 vs. 119.9 ± 11.3, *p* = 0.013). The scores for the TCBI, PNI, and HALP were similar in all groups. Similarly, the levels of the NLR, PLR, MLR, SII, SIRI, and PIV did not show significant differences between the groups. The levels of 25-OH Vitamin D and the ions Na, K, and Ca were also similar between the groups. However, magnesium levels [2 (1.9–2) vs. 2.1 (1.9–2.1) vs. 2.2 (1.96–2.2), *p* = 0.007] were significantly lower and the Na/Mg ratio [71.2 (67.4–74.7) vs. 71 (66.2–74.7) vs. 67.1 (65.2–72.5), *p* = 0.009] was significantly higher in the osteoporosis group compared to the osteopenia and control groups. Additionally, the Ca/Mg ratio was observed to be lower in the osteoporosis group compared to the control group [4.57 (4.33–4.95) vs. 4.84 (4.52–5.17), *p* = 0.045].

**Figure 1:**
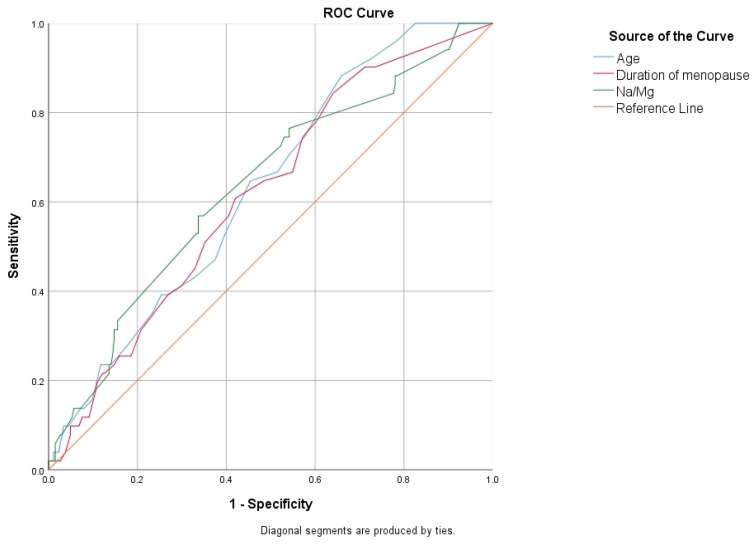
Receiver operating characteristic (ROC) curves to evaluate the usefulness of age, duration of menopause, and Na/Mg in differentiating osteoporosis.

**Figure 2:**
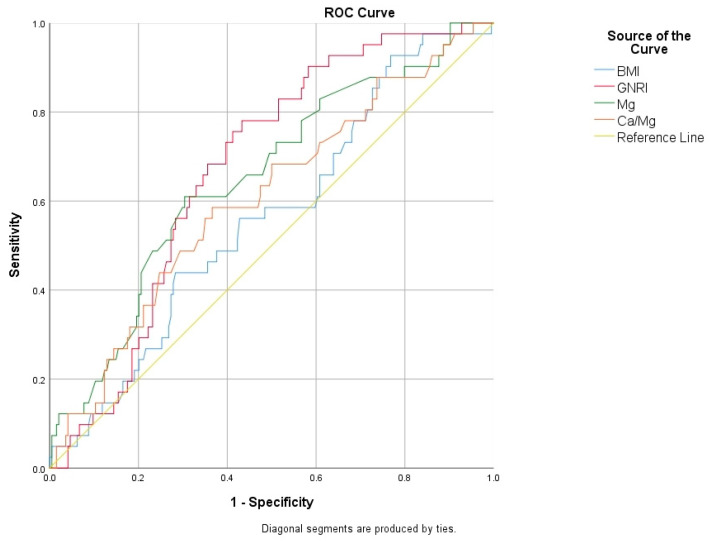
Receiver operating characteristic (ROC) curves to evaluate usefulness of BMI, GNRI, Mg, and Ca/Mg in differentiating osteoporosis.

Receiver operating characteristic (ROC) curves were calculated to evaluate the usefulness of demographic, clinical, and laboratory variables in distinguishing osteoporosis, as shown in Table 3. Maternal age demonstrated the predictive value for osteoporosis, with an area under the curve (AUC) of 0.627, a 95% confidence interval (CI) of 0.556–0.698, and a cut-off value of 56.5 years, yielding a sensitivity of 65.6% and a specificity of 53.8% (*p* = 0.002). The BMI showed an AUC of 0.563 (95% CI: 0.471–0.656), with a cut-off value of 28.1 kg/m², yielding a sensitivity of 45% and a specificity of 44.8% (*p* = 0.047). The duration of menopause displayed an AUC of 0.624 (95% CI: 0.551–0.698), with a cut-off value of 7.5 years resulting in a sensitivity of 60.7% and a specificity of 58.3% (*p* = 0.002). The GNRI score also exhibited predictive potential, with an AUC of 0.674 (95% CI: 0.597–0.752) and a cut-off value of 116.6, which achieved a sensitivity of 58.9% and a specificity of 58.3% (*p* = 0.002). Magnesium levels also showed predictive value, with an AUC of 0.652 (95% CI: 0.560–0.743) and a cut-off value of 1.98 mEq/L, yielding a sensitivity of 72.5% and a specificity of 54.3% (*p* = 0.002). Additionally, the Na/Mg ratio had an AUC of 0.635 (95% CI: 0.552–0.719), with a cut-off value of 69.7 providing a sensitivity of 59.5% and a specificity of 53.6% (*p* = 0.002). The Ca/Mg ratio exhibited an AUC of 0.606 (95% CI: 0.510–0.701), with a cut-off value of 4.69 resulting in a sensitivity of 49.8% and a specificity of 49.3% (*p* = 0.033) (Figure 1 and Figure 2).

The relationship between BMD (T-score) and clinical-laboratory variables is shown in Table 4. Maternal age (r = −0.168, *p* = 0.001), parity (r = −0.164, *p* = 0.014), duration of menopause (r = −0.173, *p* = 0.001), STRAW stage (r = −0.145, *p* = 0.005), and total cholesterol (r = −0.130, *p* = 0.013) were negatively correlated with the T-score. In contrast, the T-score showed a significant positive correlation with the BMI (r = 0.213, *p* < 0.001) and triglycerides (r = 0.209, *p* < 0.001).

The relationship between BMD (T-score) and nutritional and inflammatory indexes, the 25-OH Vitamin D level, and minerals is shown in Table 5. Mg (r = 0.155, *p* = 0.006) and the Ca/Mg ratio (r = 0.116, *p* = 0.049) showed a positive correlation with the T-score. Additionally, positive correlations were observed with the GNRI score (r = 0.179, *p* = 0.001) and TCBI score (r = 0.111, *p* = 0.033) in relation to the T-score. The Na/Mg ratio (r = −0.179, *p* = 0.005) demonstrated a significant negative correlation with the T-score.

## 4. Discussion

In this study, inflammation and nutritional indices, the 25-OH Vitamin D level, mineral levels, and their ratios were evaluated in postmenopausal women, and their relationships with BMD results were examined. Among the inflammation and nutrition scores (NLR, PLR, SII, SIRI, PIV, GNRI, TCBI, PNI, HALP) examined in this study, only the GNRI differed between the osteoporosis and control groups, and it was observed to be lower in the osteoporosis group than in the control group. In addition, among the indices examined, only the GNRI and TCBI were found to be positively correlated with the T-score. The Mg level was lower in the osteoporosis group than in the control group and osteopenia group, and the Na/Mg ratio was higher. The Ca/Mg ratio was lower in the osteoporosis group than in the control group. The T-score was positively correlated with Mg and Ca/Mg, while the Na/Mg ratio showed a significant negative correlation. Vitamin D, other minerals, and their ratios did not show significant differences between the groups.

PMOP is a significant global public health concern, affecting millions of women worldwide [28]. Early detection and prevention are crucial in managing this condition, as it can lead to severe complications such as fractures, reduced mobility, and decreased quality of life. Identifying reliable biomarkers for PMOP is important to improve early diagnosis and implement preventive measures. The use of simple, cost-effective biomarkers can help healthcare providers identifying women at risk of developing PMOP before significant bone loss occurs. Early intervention can then lead to more effective prevention strategies, including lifestyle modifications, dietary adjustments, and pharmacological treatments tailored to individual risk profiles. Therefore, our study focused on some systemic inflammation and nutritional indices, the 25-OH Vitamin D level, mineral levels, and mineral ratios.

The GNRI was first reported by Bouillanne et al. in 2005 to measure the risk of nutrition-related disease and death in elderly patients admitted to a geriatric hospital [16]. They defined the GNRI not as an index of malnutrition, but as a “nutrition-related” risk index. The GNRI is calculated based on serum albumin levels and weight loss, both of which are strong independent risk factors for morbidity and mortality in older adults. This index was found to be more effective than using these parameters individually [16]. In Bouillanne et al.’s study, serum albumin alone significantly predicted mortality only in the cases of severe malnutrition, while weight alone was not very effective unless ideal weight was considered, and it was influenced by hydration status. If the prevalence of major nutrition-related risk was determined based on albumin or the BMI alone, only 65% and 46% of patients identified using the GNRI, respectively, would be screened [16]. For this reason, they published the GNRI as a much more sensitive and specific index. Since our study included older women in the menopausal period, we thought this index might be appropriate. We observed that the GNRI score was significantly lower in the osteoporosis group and that the GNRI was positively correlated with the T-score. In our study, the predictability of the GNRI for osteoporosis was stronger compared to albumin and the BMI alone, which were not significant. Supporting our findings, Huang et al., using National Health and Nutrition Examination Survey (NHANES) data, observed a higher prevalence of osteoporosis in the adult population in the low-GNRI group compared to the high-GNRI group, and multifactorial logistic regression analysis identified a low GNRI score as an independent risk factor for osteoporosis [29]. Wang et al. isolated postmenopausal women with osteoporosis from NHANES data and showed that the GNRI was positively correlated with femoral BMD. Their study showed that each unit of increasing GNRI value was associated with a 4.13% decrease in the risk of osteoporosis [30]. Tsutsui et al. studied postoperative mortality after hip fractures in 623 Japanese patients aged ≥ 60 years. In their study, GNRI scores were classified into four risk categories: major risk (GNRI, <82), intermediate risk (82–91), low risk (92–98), and no risk (>98). Patients with major and intermediate risk for the GNRI had significantly lower overall survival [31]. Qing et al. reported in their study on elderly Chinese people that multiple regression analysis showed that GNRIs were independently associated with the total hip T-score in both genders [32]. The GNRI functions as a quantitative measure for evaluating the nutritional status of elderly people. The maintenance of a nutritious diet plays a crucial role in the regulation of bone metabolism. On the other hand, there exists a correlation between malnutrition and an increased susceptibility to the development of osteoporosis and fragility fractures [16]. The consumption of a high-quality diet has been associated with a decrease in inflammation [33,34,35] and a lower likelihood of fractures [36] among older adults. These results emphasize the value of the GNRI as an efficient and practical tool for assessing both nutritional status and bone health in postmenopausal women.

The TCBI was first used in 2018 by Doi et al. to predict adverse outcomes in the population with coronary artery disease [17]. In their study, increasing the TCBI was only slightly associated with an increased risk of all-cause and cardiovascular mortality. Additionally, to address the utility of the TCBI as a nutritional index, this study evaluated the correlation between the TCBI and GNRI, and both indices were found to be moderately positively correlated. Kim et al. evaluated GNRI and TCBI scores on mortality in acute myocardial infarction patients and showed that the GNRI was a significantly higher predictor of mortality than the TCBI [37]. In our study, we investigated the effectiveness of the TCBI score in predicting osteoporosis. The results showed that the TCBI score failed to predict osteoporosis and performed worse than the GNRI. However, we did observe a positive correlation between the TCBI score and the T-score. This suggests that while the TCBI may have some association with bone health, it is not a reliable standalone predictor of osteoporosis compared to the GNRI.

While calcium and Vitamin D are well known for their roles in maintaining bone health, magnesium is an often overlooked yet essential mineral in this context. Despite its importance, magnesium is often underemphasized in discussions about bone health. Research shows that individuals with osteoporosis often have lower serum magnesium levels compared to those with normal bone density [18,38]. Our study supports this finding, showing that magnesium levels were significantly lower in the osteoporosis group compared to the control and osteopenia groups. In contrast, the levels of other minerals such as Na, K, Ca, and the 25-OH Vitamin D level did not show any significant differences between the groups. The biological mechanisms underlying magnesium’s role in bone health are multifaceted. Magnesium contributes to the structural development of bone, influences the activity of osteoblasts and osteoclasts, and helps regulate calcium and Vitamin D metabolism [38]. Adequate magnesium intake is essential for maintaining BMD, and magnesium deficiency can lead to impaired bone formation and increased bone resorption. Clinical studies have demonstrated that magnesium supplementation can improve bone density in individuals with low magnesium levels. A randomized controlled trial by Sojka and Weaver found that postmenopausal women who received magnesium supplements experienced increased BMD compared to those who received a placebo [39]. Another study by Aydin et al. showed that magnesium supplementation in osteoporotic patients led to significant improvements in markers of bone health [40]. These findings suggest a potential role for magnesium in the prevention and treatment of osteoporosis.

Mineral ratios are calculated by dividing one mineral level by a second mineral level. Although individual mineral levels are important, the ratios between them often provide more insight into nutritional deficiencies and excesses [41]. This is because imbalances in mineral ratios can have a more significant impact on health than the absolute levels of the minerals themselves. For this reason, we investigated the relationship between the ratios of the minerals (Na/K, Na/Ca, Na/Mg, K/Mg, Ca/K, Ca/Mg) and PMOP. Among these ratios, only the Na/Mg ratio was significantly higher in the osteoporosis group than in the control group and osteopenia group. Conversely, the Ca/Mg ratio was lower in the PMOP group than in the control group. This is consistent with findings from studies indicating that a balanced Ca/Mg ratio is crucial for bone health. A study by Rude et al. demonstrated that magnesium deficiency could lead to alterations in calcium metabolism and bone structure, emphasizing the importance of an optimal Ca/Mg ratio for maintaining bone integrity [42]. Fouhy et al. stated that a Ca/Mg intake ratio between 2.2 and 3.2 could be protective against osteoporosis [43]. The significant differences in the Na/Mg and Ca/Mg ratios between the osteoporosis and control groups suggest that maintaining balanced mineral ratios is significant for bone health.

This study has some limitations. Our study found that the GNRI was significantly lower in individuals with osteoporosis compared to controls. This finding suggests that the GNRI may have potential as an indicator for assessing bone health, although it is important to note that this association does not necessarily imply causation. The observed differences could result from other unmeasured factors beyond osteoporosis itself. Further studies are warranted to explore the relationship between the GNRI, nutritional status, and bone health in greater detail, with a particular focus on excluding confounding variables. This study’s data collection was limited to a single time point, meaning that nutritional data such as serum albumin and weight were recorded only once for all participants. Similarly, total BMD-T values (for the lumbar spine and femoral neck) were measured only once; this may introduce potential bias in GNRI and T-scores. Additionally, screening profile X-rays of the spine were not performed to identify asymptomatic vertebral fractures, which may have been prevalent among the participants. This omission limits our ability to fully assess the clinical burden of osteoporosis in this cohort. Furthermore, data regarding participants’ alcohol and tobacco consumption as well as Vitamin D supplement use are missing in our study. These factors can significantly affect BMD and osteoporosis risk, and ignoring them is a notable limitation. To address these limitations, it is imperative to conduct multicenter clinical trials. Such studies will confirm our findings and provide a systematic evaluation of changes in BMD and each variable that may affect osteoporosis. Extended monitoring is also necessary to explore the precise impact of nutritional levels on the onset and progression of osteoporosis in postmenopausal women. By tracking these factors over time, researchers can better understand the dynamic relationship between nutrition and bone health, ultimately leading to more effective prevention and treatment strategies.

## 5. Conclusions

This study showed that among the inflammatory and nutritional scores (NLR, PLR, SII, SIRI, PIV, GNRI, TCBI, PNI, HALP), only the GNRI differed between the osteoporosis and control groups, and it was observed to be lower in the osteoporosis group than in the control group. Magnesium levels were notably lower in the osteoporosis group compared to the control and osteopenia groups, while the Na/Mg ratio was higher and the Ca/Mg ratio was lower in the osteoporosis group. Our findings suggest that the GNRI could serve as a useful indicator for assessing bone health and the risk of osteoporosis. Furthermore, maintaining appropriate levels of magnesium and balanced Na/Mg and Ca/Mg ratios appears crucial for bone density. Future studies, particularly multicenter clinical trials, are needed to validate these results and further explore the dynamic relationship between nutritional factors and osteoporosis, ultimately guiding more effective prevention and treatment strategies for postmenopausal women.

## Figures and Tables

**Table 1:** Demographic, clinical, and laboratory characteristics and the outcomes between the groups.

	Osteoporosis Group n: 61	Osteopenia Group n: 153	Control Group n: 154	*p*-Value
Age (year)	61 ± 9	57 ± 8	55 ± 8	<0.001 ^a^
Gravida	4 ± 3	3 ± 2	3 ± 2	0.540
Parity	3 ± 2	2 ± 1	2 ± 1	0.107
BMI (kg/m^2^)	27.7 ± 35	28.5 ± 4.7	29.8 ± 5.6	0.029 ^b^
Age of menopause (year)	48 ± 4	48 ± 5	48 ± 4	0.485
Duration of menopause (year)	12 ± 10	9 ± 9	7 ± 8	0.001 ^b^
STRAW stage				0.008
1a	5 (8.2%)	34 (22.2%)	51 (33.1%)	
1b	3 (4.9%)	10 (6.5%)	12 (7.8%)	
1c	12 (19.7%)	27 (17.6%)	23 (14.9%)	
2	41 (67.2%)	82 (53.6%)	68 (44.2%)	
Bone mineral density (T-score)	−2.98 ± 0.44	−1.72 ± 0.46	0.21 ± 1.02	<0.001
Albumin (g/dL)	4.30 ± 0.27	4.24 ± 0.28	4.24 ± 0.29	0.450
Total cholesterol (mg/dL)	221.6 (183.9–259.8)	221.1 (190.1–254)	209.7 (191.7–236.7)	0.170
Triglyceride (mg/dL)	137 (89.5–169)	121 (83–171)	128 (88–186)	0.649
Hemoglobin (g/dL)	13.4 ± 1	13.3 ± 1.1	13.4 ± 1.2	0.999
Neutrophil (×10^3^/uL)	3.69 (2.97–4.31)	3.55 (2.81–4.42)	3.53 (2.87–4.46)	0.905
Lymphocyte (×10^3^/uL)	1.96 (1.65–2.53)	2.11 (1.64–2.52)	2.22 (1.86–2.58)	0.131
Monocyte (×10^3^/uL)	0.39 (0.29–0.49)	0.35 (0.28–0.41)	0.36 (0.30–0.44)	0.116
Platelet (×10^3^/uL)	281 (226.5–317)	259.5 (220–300)	272 (230–309)	0.186

BMI: body mass index; STRAW: stages of reproductive aging workshop. Data are shown as mean ± SD, median (Q1–Q3), or n,%. ^a^ The differences between the control group and the osteoporosis group and between the osteopenia group and osteoporosis group are significant. ^b^ The differences between the control group and the osteoporosis group are significant.

**Table 2:** An examination of nutritional and inflammatory indexes, the 25-OH Vitamin D level, and minerals between the groups.

	Osteoporosis Group n: 61	Osteopenia Group n: 153	Control Group n: 154	*p*-Value
GNRI	116 ± 9.4	117.2 ± 9.8	119.9 ± 11.3	0.013 ^a^
TCBI	1757 (1245–2866)	1941 (1183–2865)	2040 (1165–3358)	0.535
PNI	53.5 ± 4.5	52.8 ± 4.3	53.8 ± 4.9	0.176
HALP score	42.3 (34.6–54.4)	46.9 (35.3–57.7)	45.4 (34.8–59.7)	0.508
NLR	1.70 (1.38–2.11)	1.66 (1.36–2.17)	1.59 (1.28–2.01)	0.232
PLR	134 (111.2–159)	123.3 (100–155)	125 (102–155.9)	0.351
MLR	0.17 (0.13–0.23)	0.17 (0.14–0.20)	0.16 (0.14–0.20)	0.234
SII	466.8 (363.3–617.3)	426.5 (330.9–576.4)	416.2 (333.1–581.4)	0.384
SIRI	0.64 (0.44–0.89)	0.58 (0.43–0.81)	0.57 (0.43–0.81)	0.331
PIV	169.5 (118.2–259.5)	147.3 (101.1–226)	152.9 (108.7–216)	0.166
25-OH Vitamin D (ng/mL)	24.11 ± 11.51	26.51 ± 17.39	22.53 ± 10.80	0.438
Na (mEq/L)	139.1 ± 2.9	139.3 ± 2.3	139.4 ± 2.6	0.751
K (mEq/L)	4.32 ± 0.26	4.33 ± 0.34	4.26 ± 0.35	0.344
Ca (mEq/L)	9.50 (9.30–9.75)	9.50 (9.30–9.82)	9.50 (9.20–9.70)	0.525
Mg (mEq/L)	2 (1.9–2)	2.1 (1.9–2.1)	2.2 (1.96–2.2)	0.007 ^b^
Na/K	32.2 ± 2	32.4 ± 2.5	32.9 ± 2.8	0.430
Na/Ca	14.6 ± 0.8	14.7 ± 0.7	14.8 ± 0.7	0.226
Na/Mg	71.2 (67.4–74.7)	71 (66.2–74.7)	67.1 (65.2–72.5)	0.009 ^b^
K/Mg	2.1 (2–2.26)	2.19 (2.05–2.37)	2.15 (1.95–2.39)	0.263
Ca/K	2.21 ± 0.16	2.21 ± 0.19	2.23 ± 0.22	0.907
Ca/Mg	4.57 (4.33–4.95)	4.80 (4.52–5.11)	4.84 (4.52–5.17)	0.045 ^a^

GNRI: geriatric nutritional risk index; TCBI: triglycerides, total cholesterol, and body weight index; PNI: prognostic nutritional index; HALP: hemoglobin, albumin, lymphocyte, and platelet score; NLR: neutrophil-to-lymphocyte ratio; PLR: platelet-to-lymphocyte ratio; MLR: monocyte-to-lymphocyte ratio; SII: systemic immune inflammation index; SIRI: systemic inflammation response index; PIV: pan-immune inflammation value. Data are shown as mean ± SD or median (Q1–Q3). ^a^ The differences between the control group and the osteoporosis group are significant. ^b^ The differences between the control group and the osteoporosis group and between the osteopenia group and osteoporosis group are significant.

**Table 3:** Receiver operating characteristic (ROC) curves to evaluate the usefulness of demographic, clinical, and laboratory variables in differentiating osteoporosis.

	AUC	95% CI	Cut-Off Value	Sensitivity (%)	Specificity (%)	*p*-Value
Age (year)	0.627	0.556–0.698	56.5	65.6	53.8	0.002
BMI (kg/m^2^)	0.563	0.471–0.656	28.1	45	44.8	0.047
Duration of menopause (year)	0.624	0.551–0.698	7.5	60.7	58.3	0.002
GNRI	0.674	0.597–0.752	116.6	58.9	58.3	0.002
Mg (mEq/L)	0.652	0.560–0.743	1.98	72.5	54.3	0.002
Na/Mg	0.635	0.552–0.719	69.7	59.5	53.6	0.002
Ca/Mg	0.606	0.510–0.701	4.69	49.8	49.3	0.033

BMI: body mass index; GNRI: geriatric nutritional risk index.

**Table 4:** Relationship between bone mineral density (T-score) and clinical-laboratory variables.

	r	*p*-Value
Age (year)	−0.168	0.001
Gravida	−0.094	0.178
Parity	−0.164	0.014
BMI (kg/m^2^)	0.213	<0.001
Age of menopause (year)	0.027	0.606
Duration of menopause (year)	−0.173	0.001
STRAW stage	−0.145	0.005
Albumin (g/dL)	−0.072	0.168
Total cholesterol (mg/dL)	−0.130	0.013
Triglyceride (mg/dL)	0.209	<0.001
Hemoglobin (g/dL)	0.022	0.675
Neutrophil (×10^3^/uL)	−0.010	0.843
Lymphocyte (×10^3^/uL)	0.099	0.059
Monocyte (×10^3^/uL)	−0.011	0.829
Platelet (×10^3^/uL)	0.039	0.454

BMI: body mass index; STRAW: stages of reproductive aging workshop.

**Table 5:** Relationship between bone mineral density (T-score) and nutritional and inflammatory indexes, 25-OH Vitamin D level, and minerals.

	r	*p*-Value
GNRI	0.179	0.001
TCBI	0.111	0.033
PNI	0.045	0.386
HALP score	0.035	0.504
NLR	−0.036	0.500
PLR	−0.044	0.407
MLR	−0.063	0.236
SII	−0.039	0.462
SIRI	−0.030	0.576
PIV	−0.033	0.540
25-OH Vitamin D (ng/mL)	−0.063	0.352
Na (mEq/L)	0.040	0.518
K (mEq/L)	−0.059	0.334
Ca (mEq/L)	−0.060	0.289
Mg (mEq/L)	0.155	0.006
Na/K	0.075	0.227
Na/Ca	0.078	0.223
Na/Mg	−0.179	0.005
K/Mg	−0.019	0.757
Ca/K	0.047	0.449
Ca/Mg	0.116	0.049

GNRI: geriatric nutritional risk index; TCBI: triglycerides, total cholesterol, and body weight index; PNI: prognostic nutritional index; HALP: hemoglobin, albumin, lymphocyte, and platelet score; NLR: neutrophil-to-lymphocyte ratio; PLR: platelet-to-lymphocyte ratio; MLR: monocyte-to-lymphocyte ratio; SII: systemic immune inflammation index; SIRI: systemic inflammation response index; PIV: pan-immune inflammation value.

## Data Availability

Upon reasonable request, the corresponding author will provide the information supporting this study’s conclusions.
